# A real-world study assessing the impact of retinal fluid on visual acuity outcomes in patients with neovascular age-related macular degeneration in Korea

**DOI:** 10.1038/s41598-022-18158-z

**Published:** 2022-08-19

**Authors:** Jae Hui Kim, Min Sagong, Se Joon Woo, Yu Cheol Kim, Heeyoon Cho, Young Hoon Lee, Iksoo Byon, Young Joon Jo, Hee Seung Chin, Youkyung Lee, Jae Eun Chae, Se Woong Kang

**Affiliations:** 1 Department of Ophthalmology, Kim’s Eye Hospital, Seoul, Korea; 2 Department of Ophthalmology, Yeungnam University College of Medicine, Daegu, Korea; 3 Department of Ophthalmology, Seoul National University Bundang Hospital, Seoul National University College of Medicine, Seongnam, Korea; 4 Department of Ophthalmology, Keimyung University School of Medicine, Daegu, Korea; 5 Department of Ophthalmology, Hanyang University Guri Hospital, Hanyang University College of Medicine, Guri, Korea; 6 Department of Ophthalmology, Konyang University College of Medicine, Daejeon, Korea; 7 Department of Ophthalmology, Pusan National University Hospital, Pusan National University School of Medicine, Busan, Korea; 8 Department of Ophthalmology, Chungnam National University Hospital, Daejeon, Korea; 9 Department of Ophthalmology, Inha University School of Medicine, Incheon, Korea; 10 Clinical Development and Medical Affairs, Novartis Korea, Seoul, Korea; 11 LSK Global Pharma Services, Seoul, Korea; 12 Department of Ophthalmology, Samsung Medical Center, Sungkyunkwan University School of Medicine, 81 Irwon-Ro, Gangnam-Gu, Seoul, 06351 Korea

**Keywords:** Corneal diseases, Macular degeneration

## Abstract

To evaluate the real-world treatment outcomes in patients with neovascular age-related macular degeneration (nAMD) in Korea, focusing on retinal fluid resolution. This multi-institutional retrospective chart review study, analyzed medical records of patients with nAMD (age ≥ 50 years) who received their first anti-vascular endothelial growth factor (VEGF) treatment in ophthalmology clinics across South Korea between January 2017 and March 2019. The primary endpoint was the proportion of patients with retinal fluid after 12 months of anti-VEGF treatment. The association between fluid-free period and VA gains was also evaluated. A total of 600 patients were enrolled. At baseline, 97.16% of patients had retinal fluid; after 12 months of anti-VEGF treatment, 58.10% of patients had persistent retinal fluid. VA improvements were relatively better in patients with absence of retinal fluid compared with presence of retinal fluid (+ 12.29 letters vs. + 6.45 letters at month 12; *P* < .0001). Longer duration of absence of retinal fluid over first 12 months correlated with better VA gains at month 12 (*P* < .01). More than half of the study patients with nAMD had retinal fluid even after 12 months of treatment with their current anti-VEGF. Presence of retinal fluid was associated with relatively worse VA outcomes.

## Introduction

Age-related macular degeneration (AMD) is the most common cause of irreversible vision loss in the elderly^1^. A nationwide population-based study using data from the Korean National Health Claims database found that neovascular AMD (nAMD) had a prevalence of 36.43 per 10,000 people and an incidence of 3.02 per 10,000 person-years in people over the age of 40 years^2^. nAMD is associated with limitations in both mobility and activity as a result of vision loss, leading to a negative impact on the quality of life of patients^3^. The use of anti-vascular endothelial growth factor (anti-VEGF) agents as a standard of care for nAMD has resulted in a significant decrease in the incidence of blindness and visual impairment^4^.

In Korea, ranibizumab (Lucentis; Novartis Pharma, Basel, Switzerland; Genentech, South San Francisco, CA, USA) and aflibercept (Eylea; Regeneron Pharma, Tarrytown, NY, USA; Bayer, Basel, Switzerland) are approved anti-VEGFs to treat visual impairment due to nAMD^5^. Off-label use of bevacizumab (Avastin; Genentech) for nAMD is also common in Korea due to cost issue^6^. Despite remarkable success in clinical trials, the 2 major challenges in the application of anti-VEGF therapy in real-world practice are the high treatment burden, requiring frequent hospital visits and injections, and the associated high costs^7^. As a result, patients with nAMD usually have suboptimal long-term outcomes with anti-VEGF therapy in real-world settings; this might be due to delays in diagnosis and/or treatment approval and initiation, differences in individual patient response to different anti-VEGF therapies, lapses in physician regimentation of anti-VEGF injection and monitoring, and inadequate patient adherence to treatment and monitoring^7–11^.

Morphological signs of disease activity on optical coherence tomography (OCT) are crucial because they correspond to early signs of recurrence, which are usually observed prior to visual acuity (VA) loss^12^.Central subfield thickness (CST) and fluid status, measured by OCT, are frequently used by clinicians as the key indicators of disease activity and for driving treatment decisions^13,14^. Retinal fluid is an important parameter in nAMD disease activity, and one of the objectives of nAMD treatment is to achieve complete fluid resolution. The presence of fluid is a key retreatment criterion across the landmark clinical trials for nAMD, as well as in current major clinical practice guidelines globally^13–16^.

The aim of this study was to better understand the real-world treatment patterns and outcomes of patients with nAMD in Korea, with a focus on retinal fluid resolution with current anti-VEGF therapies.

## Results

### Patient disposition

**Figure 1:**
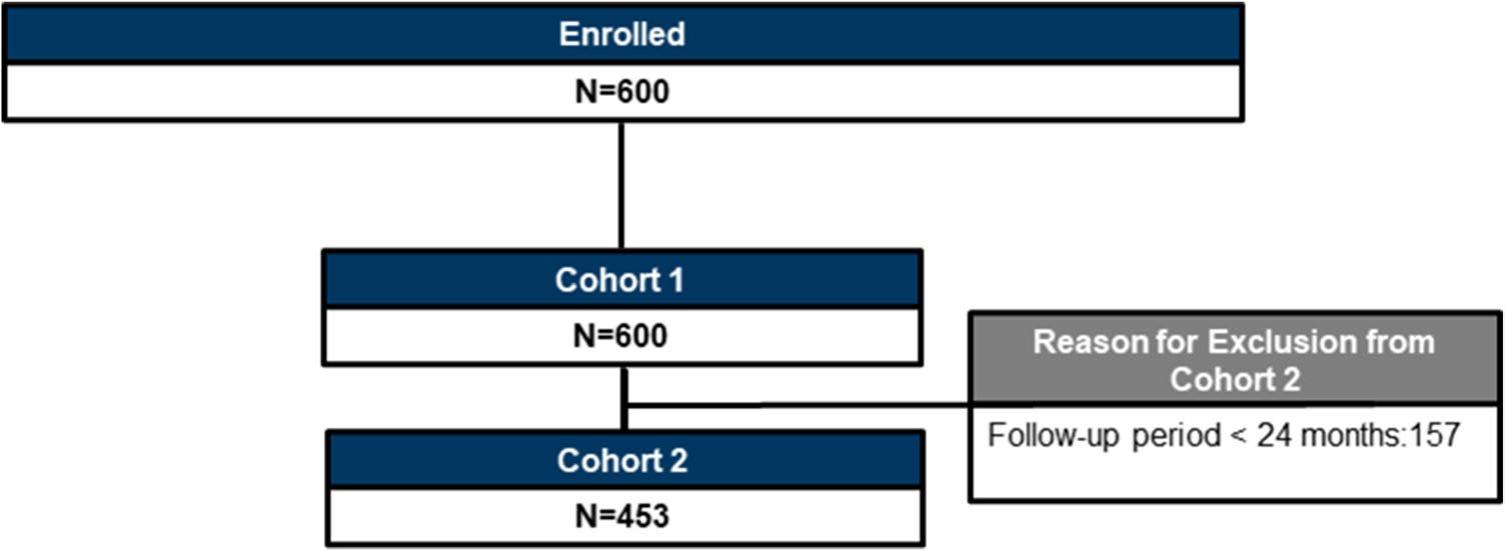
Patient disposition. Cohort 1: Patients who met the eligibility criteria and had a minimum follow-up period of 12 months. Cohort 2: Patients who met the eligibility criteria with a minimum follow-up period of 24 months. N, total number of patients.

A total of 600 patients were enrolled; cohort 1 included all 600 patients who had a minimum follow-up period of 12 months, and cohort 2 included 453 patients who had a minimum follow-up period of 24 months (Fig. 1).

### Patient demographic and baseline clinical characteristics

The mean (SD) age of the study patients was 73.33 (8.88) years, and a higher proportion of the patients were male (57.83%). At baseline, a majority of the patients (67.83%) had subfoveal lesions; 28.33% and 9.5% of patients had polypoidal choroidal vasculopathy and retinal angiomatous proliferation, respectively (Table 1).

**Table 1:** Patient demographics and characteristics (Cohort 1).

Baseline characteristics	*N* = 600
**Demographics**	
**Age (years)**	
Mean (SD)	73.33 (8.88)
**Sex, *n* (%)**	
Male	347 (57.83)
**Clinical characteristics (study eye)**	
**CNV lesion location, *n* (%)**	
Extrafoveal	48 (8.00)
Juxtafoveal	145 (24.17)
Subfoveal	407 (67.83)
**Total lesion size, *n* (%)**	
≤ 1 DA	204 (34.00)
1–4 DA	330 (55.00)
> 4 DA	63 (10.50)
**CNV lesion composition, *n* (%)**	
Predominantly classic	162 (27.00)
Minimally classic	61 (10.17)
Occult	372 (62.00)
**PCV, *n* (%)**	
Yes	170 (28.33)
No	387 (64.50)
Un-assessable	43 (7.17)
**RAP, *n* (%)**	
Yes	57 (9.50)
No	502 (83.67)
Un-assessable	41 (6.83)
Index anti-VEGF therapy, *n* (%)^*^	
Ranibizumab	211 (35.16)
Bevacizumab	104 (17.34)
Aflibercept	285 (47.5)

CNV, choroidal neovascularization; DA, disc area; n, number of patients; N, total number of patients; PCV, polypoidal choroidal vasculopathy; RAP, retinal angiomatous proliferation; SD, standard deviation.

*Index anti-VEGF, anti-VEGF injection taken during the first/baseline visit.

### Treatment pattern

The mean (95% CI) number of injections and visits during the first year of treatment was 5.54 (5.36, 5.72) and 9.51 (9.32, 9.71), respectively. A similar proportion of patients were treated as per the treat and extend (T&E; 50.5%) and *pro re nata* (PRN; 43.8%) regimens. During the second year, the mean (95% CI) number of injections and visits was 3.48 (3.26, 3.73) and 7.10 (6.86, 7.36), respectively, and 49.0% and 45.0% of patients were treated as per the T&E and PRN treatment regimens, respectively. Approximately 6% of patients were treated as per “Other” treatment regimens, which included a hybrid of T&E and PRN. During the first year, 417 patients (69.5%) were treated using single anti-VEGF agent, while the remaining 183 patients (30.5%) underwent treatment switch. During the second year, 213 patients of the 453 (47.02%) in the cohort having a follow-up of 24 months remained on single anti-VEGF, while 150 (33.11%) underwent treatment switch (Table 2).

**Table 2:** Treatment pattern during the first and second year of anti-VEGF treatment.

	All
**First year of treatment**	(N = 600)
*n*	600
Mean number of injections (95% CI)	5.54 (5.36,5.72)
Mean number of non-injection visits (95% CI)	3.97 (3.78,4.18)
Mean total number of visits (95% CI)	9.51 (9.32,9.71)
**Treatment regimen, *n* (%)**	
T&E	303 (50.5)
PRN	263 (43.8)
Others	34 (5.7)
Patients staying on index therapy, *n* (%)	417 (69.5)
Patients switching therapy, *n* (%)	183 (30.5)
**Second year of treatment**	(n = 573)
*n*	573
Mean number of injections (95% CI)	3.48 (3.26,3.73)
Mean number of non-injection visits (95% CI)	3.62 (3.39,3.86)
Mean total number of visits (95% CI)	7.10 (6.86,7.36)
**Treatment regimen, *n* (%)**	
T&E	281 (49.0)
PRN	258 (45.0)
Others	34 (5.9)
Patients staying on index therapy, n/N (%)^*^	213//453 (47.02)
Patients switching therapy, n/N (%)^*^	150/453 (33.11)

CI, confidence interval; N, total number of patients; n, number of patients; PRN, *pro re nata*; T&E, treat and extend; VEGF, vascular endothelial growth factor.

*Of the 573 patients who entered second year of treatment, only 453 completed 24 months follow-up. Patients staying on index therapy and those switching therapy in the second year are calculated using these 453 patients completing 24 months follow-up.

### Retinal fluid status over time

**Figure 2:**
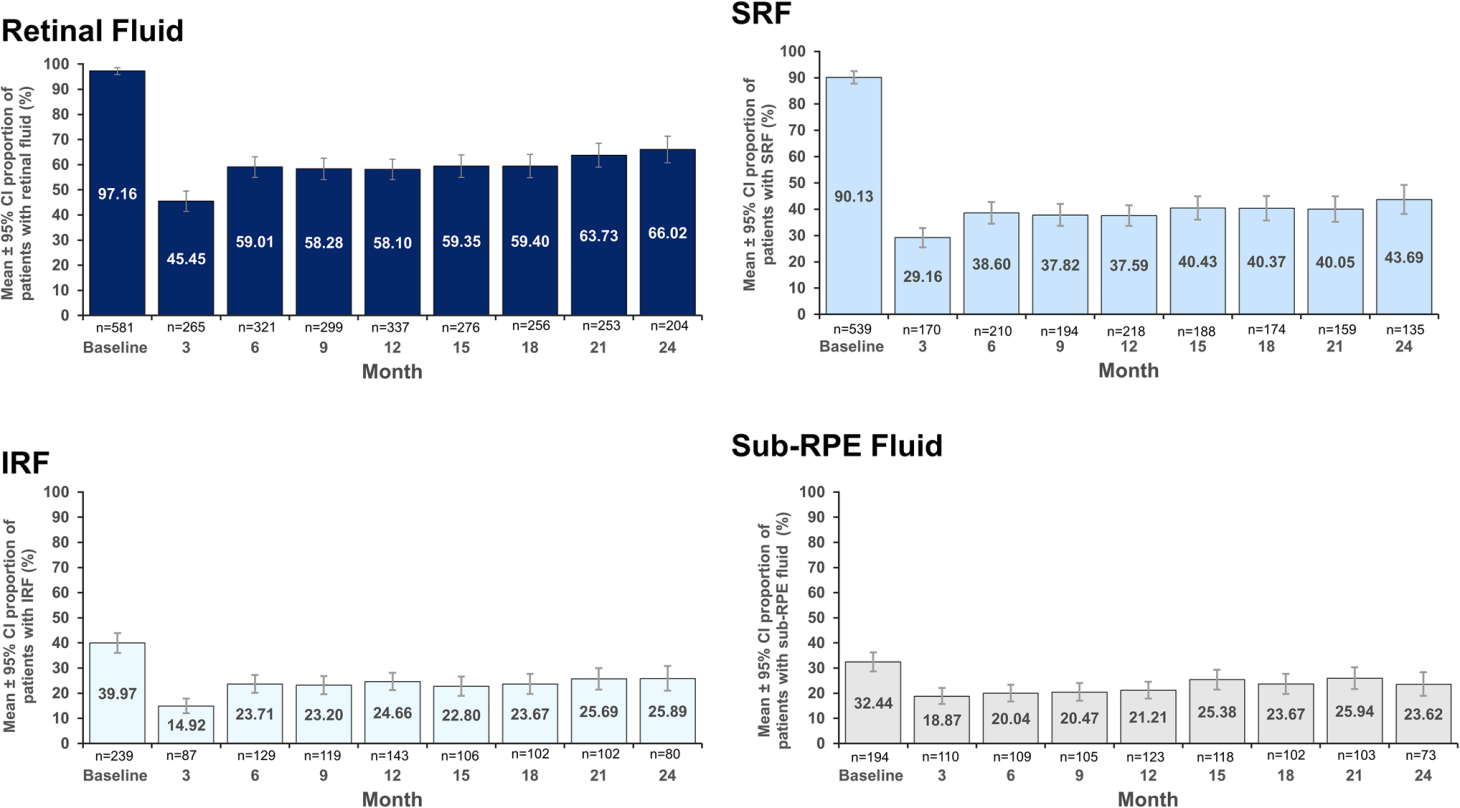
Proportion of patients with presence of retinal fluid from baseline to 24 months (cohort 1). CI, confidence interval; IRF, intraretinal fluid; RPE, retinal pigment epithelium; SRF, subretinal fluid.

Figure 2 depicts the change in the proportion of patients with retinal fluid from baseline to 24 months, and by the type of retinal fluid (IRF, SRF, and sub-RPE fluid). At baseline, 97.16% of patients had retinal fluid (IRF: 39.97%, SRF: 90.13%, and sub-RPE fluid: 32.44%). There was a decline in the proportion of patients with retinal fluid, IRF, SRF, and sub-RPE fluid up to month 24 after anti-VEGF treatment compared to baseline. The greatest decline was observed at 3 months, but thereafter a slight increase in the proportion of patients with retinal fluid between months 6 and 24 was seen. At month 12, the proportion of patients with retinal fluid were 58.10% (IRF: 24.66%, SRF: 37.59%, and sub-RPE fluid: 21.21%). At month 24, 66.02% of patients had presence of retinal fluid (IRF: 25.89%, SRF: 43.69%, and sub-RPE fluid: 23.62%) (Fig. 2).

### Visual acuity and central subfield thickness changes over time

**Figure 3:**
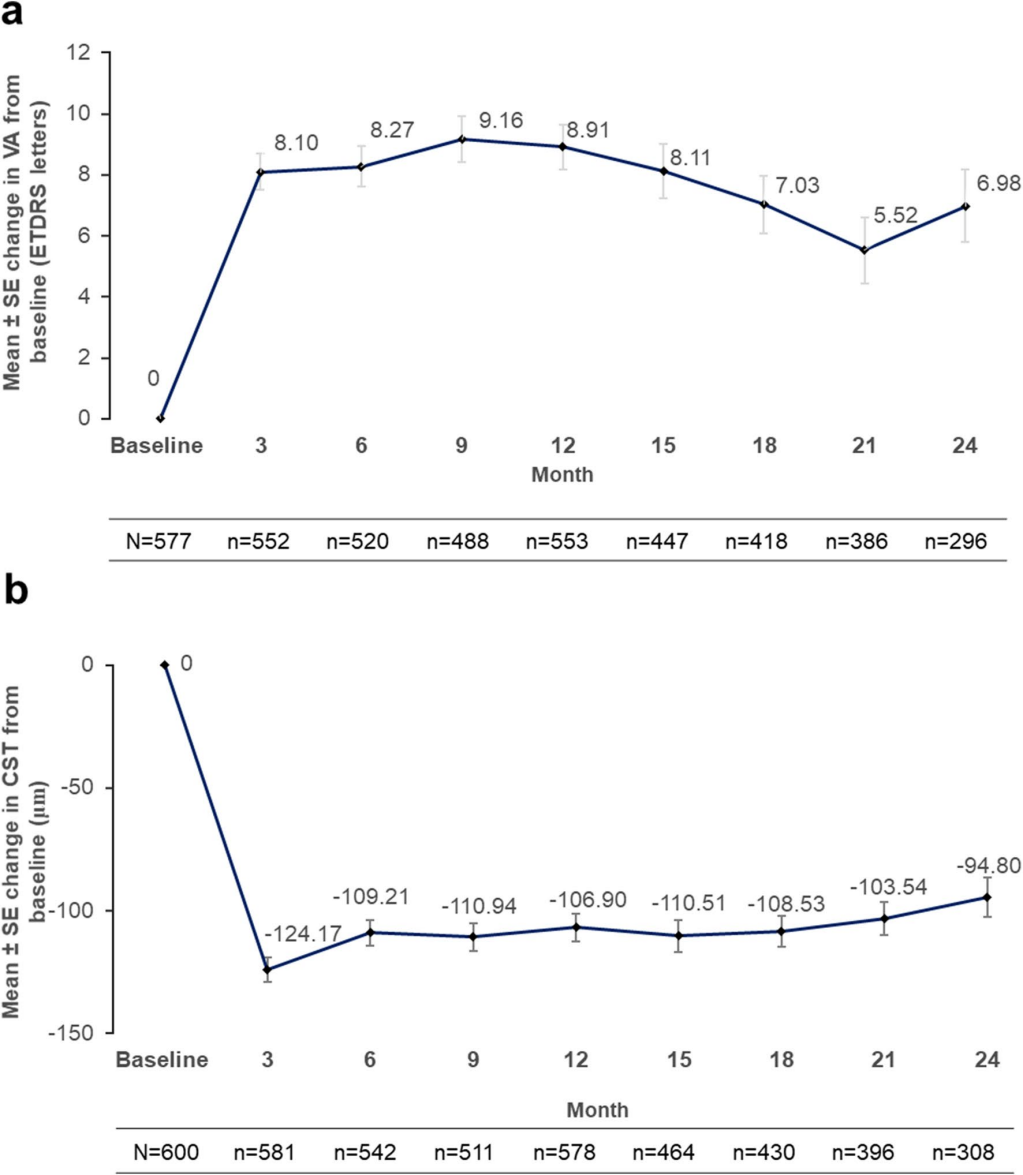
Mean change in (**a**) visual acuity and (**b**) ^*^central subfield thickness from baseline to 24 months (cohort 1). ^*^95% CI values for change in CST at each time point excludes 0, hence making the decrease in CST significant.

Following anti-VEGF treatment, the maximum gain in VA (Early Treatment Diabetic Retinopathy Study [ETDRS] letters) from baseline (mean VA: 54.14 letters) was observed at 3 months (mean: 62.03 letters, mean change: + 8.10 letters). The VA gains were maintained through 12 months (mean: 63.18 letters, mean change: + 8.91 letters) but showed a trend of gradual decline by 24 months of treatment (mean: 61.81 letters, mean change: + 6.98 letters) (Fig. 3a).

Similarly, a significant decrease in CST from baseline was observed after 3 months of anti-VEGF treatment (mean: 256.72 μm, mean change: − 124.17 μm), which remained stable over 12 months (mean: 273.00 μm, mean change: − 106.90 μm). There was a small increase in CST between months 12 and 24 (mean: 282.50 μm, mean change: − 94.80 μm) (Fig. 3b).

### Visual acuity changes by presence of retinal fluid at each time point

**Figure 4:**
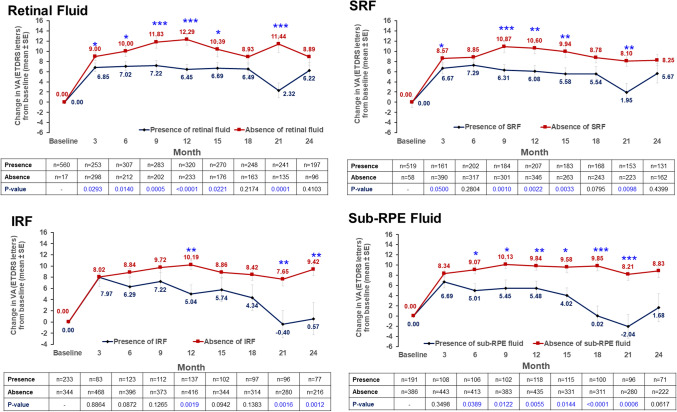
Visual acuity change by absence/presence of retinal fluid at each time point (cohort 1). ETDRS, Early Treatment Diabetic Retinopathy Study; IRF, intraretinal fluid; RPE, retinal pigment epithelium; SE, standard error; SRF, subretinal fluid; VA, visual acuity. *P value* analyzed for difference between presence and absence of retinal fluid groups (Wilcoxon signed-rank test). *P < .05, **P < .01, ***P < .001.

VA gains from baseline were significantly greater in patients with absence of retinal fluid versus those with presence of retinal fluid, with a *P value* of < 0.05 at months 3, 6, 9, 12, 15, and 21. At months 18 and 24, though VA gains were higher in the absence of retinal fluid compared to presence of fluid, the difference in between the two groups was not significant (*P value* > 0.05). At month 12, the mean difference in VA gain between patients with absence of retinal fluid versus those with presence of retinal fluid was 5.84 letters. Similarly, at any given time point, VA gains were relatively better in patients with absence of IRF, SRF, and sub-RPE fluid. (Fig. 4) The mean VA at each time point is shown in Fig. S1.

### Visual acuity changes by presence of retinal fluid at month 3

**Figure 5:**
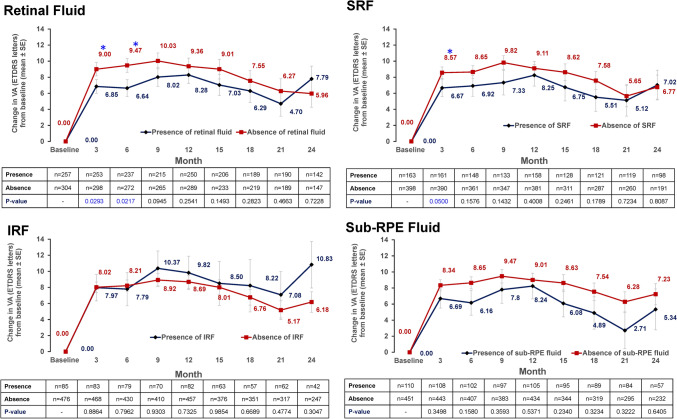
Visual acuity change by absence/presence of retinal fluid at month 3 (cohort 1). ETDRS, Early Treatment Diabetic Retinopathy Study; IRF, intraretinal fluid; RPE, retinal pigment epithelium; SE, standard error; SRF, subretinal fluid; VA, visual acuity. *P* value analyzed for difference between presence and absence of retinal fluid groups (Wilcoxon signed-rank test). *P < .05,**P < .01,***P < .001. The patient eyes were divided into the presence or absence of fluid at Month 3 and change in VA was stratified of those groups at all subsequent time points.

We assessed the VA outcome at all time points based on the presence/absence of retinal fluid and the types of retinal fluid only at month 3 (after the loading dose for anti-VEGF therapy) (Fig. 5). VA gains were relatively better in patients who achieved absence of retinal fluid at month 3 versus those with presence of retinal fluid, with a *P value* of < 0.05 at months 3 and 6. VA gains were numerically greater in patients with absence of SRF and sub-RPE fluid at month 3, from month 3 to approximately month 24. However, the difference was not enough to attain statistical significance (*P value* > 0.05). VA gains were numerically lower in patients with absence of IRF at month 3, but baseline VA was lower in patients with presence of IRF at month 3 (mean VA: 55.66 letters for absence of IRF vs 45.32 letters for presence of IRF). The mean VA at each time point is shown in Fig. S2.

### Visual acuity change by quartile time with absence of retinal fluid

**Figure 6:**
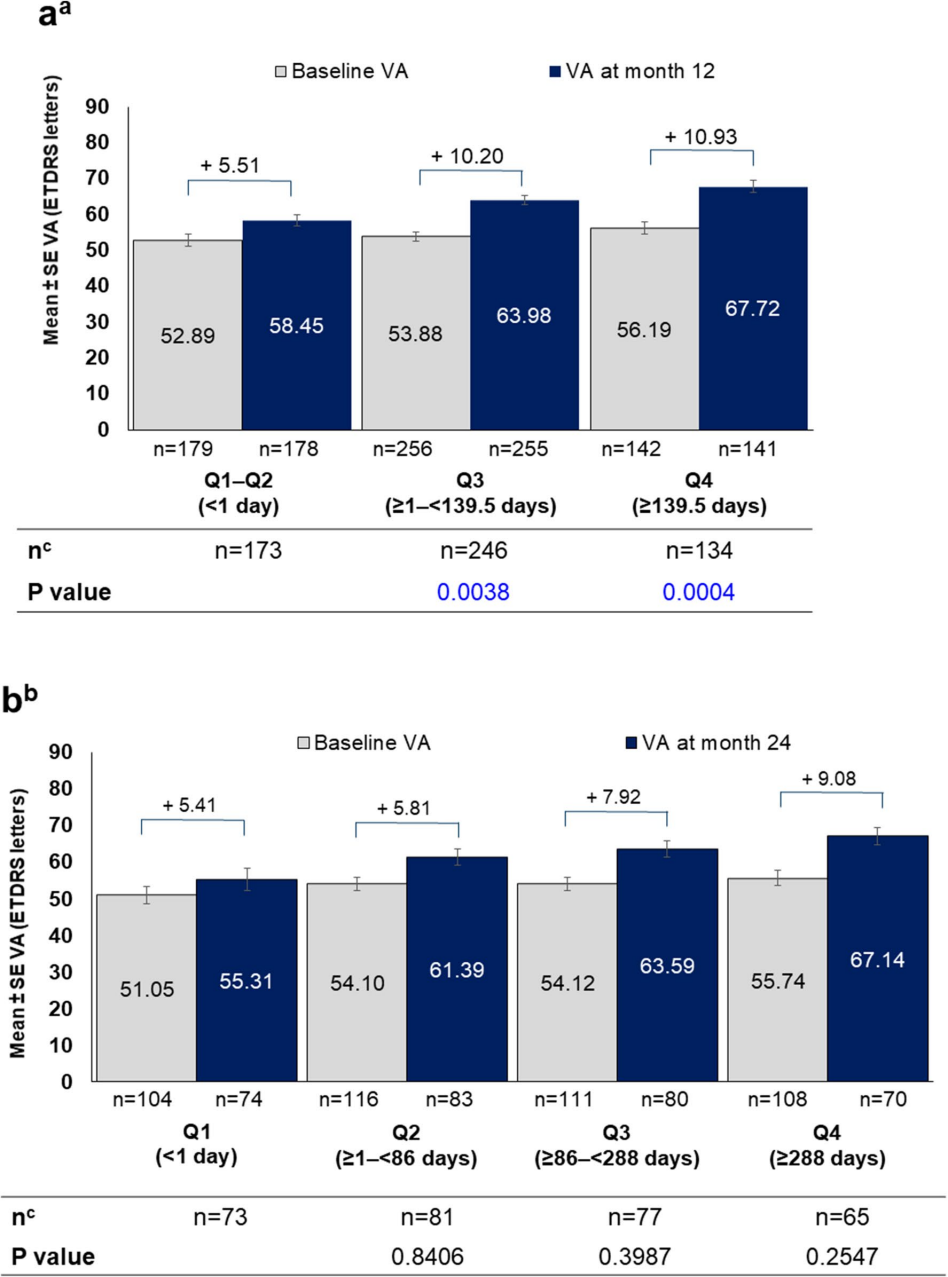
Visual acuity from baseline to month 12 and 24 stratified by quartile of time with absence of retinal fluid over (**a**) months 1 to 12 (cohort 1) and (**b**) months 1 to 24 (cohort 2). ETDRS, Early Treatment Diabetic Retinopathy Study; N, number of patients; Q, quartile; SE, standard error; VA, visual acuity. ^a^Patients' eyes were divided into quartiles groups based on time with absence of fluid over months 1 to 12, and change in VA was stratified for those quartile groups. Lower quartile = 0.0, median = 1.0, upper quartile = 139.5 days. Q1-Q2: less than median, (<1 day); Q3: median to less than upper quartile (≥ 1 day to < 139.5 days); Q4: more than or equal to upper quartile (≥ 139.5 days). P value test for difference between Q1-Q2 and each quartile group (Wilcoxon signed-rank test). ^b^Patients' eyes were divided into quartiles based on time with absence of fluid over months 1 to 24, and change in VA was stratified for those quartile groups. Lower quartile = 1.0, median = 86.0, upper quartile = 288.0 days. Q1: less than lower quartile (<1 day); Q2: lower quartile to less than median (≥ 1 to < 86 days); Q3: median to less than upper quartile (≥86 to <288 days); Q4: more than or equal to upper quartile (≥ 288 days). P value test for difference between Q1 and each quartile group (Wilcoxon signed-rank test). ^c^Number of patients with both baseline and month 12 or 24 VA; changes in VA were calculated in these patients.

After categorizing patients into quartiles based on time of absence with retinal fluid, patients with a longer duration of absence of retinal fluid from 1 to 12 months showed higher VA gains. There were substantial differences in VA gains at month 12 for the Q1–Q2 subgroup compared with Q3 (*P* = 0.0038) and Q4 subgroups (*P* = 0.0004) (Fig. 6a).

Similarly, when patients were divided into quartiles based on time of absence of retinal fluid from 1 to 24 months, VA gains at month 24 were also relatively higher in the group with a longer duration of absence of retinal fluid. However, the *P values* for the differences between the groups were > 0.05 (Fig. 6b).

## Discussion

To the best of our knowledge, the PROOF study is the first multicenter study to assess the efficacy of anti-VEGFs in resolving retinal fluid in nAMD in the real-world setting in Korea. This study found that despite their current anti-VEGF treatment, a high proportion of patients with nAMD continued to have retinal fluid at year 1 as well as year 2 of treatment. Further, patients who achieved early fluid resolution showed a trend towards better VA gains.

Disease activity in nAMD has been defined based on 3 parameters: (1) a loss of ≥ 5 letters in VA, (2) evidence of new hemorrhage, and (3) the presence of retinal fluid, including IRF and SRF^17^. Clinical practice guidelines from the American Academy of Ophthalmology^14^ and the European Society of Retina Specialists^13^ recommend retreatment with anti-VEGFs if retinal fluid is detected on OCT as it is an indication of active disease. A nAMD study from United Kingdom including 1190 eyes showed that presence of IRF and SRF has greater influence on real-world anti-VEGF retreatment decisions compared to the presence of pigment epithelial detachment or vision loss^18^.

In the present study, the reduction in the proportion of patients with retinal fluid was most noticeable by 3 months (after the loading dose), although only 54.55% of patients achieved fluid-free status. However, > 50% of the patients treated with anti-VEGFs had retinal fluid at the end of both year 1 and year 2 of the treatment. One possible reason for the high proportion of patients without resolution of retinal fluid in this study was the use of PRN regimen in nearly half of the patients. However, a subset of patients may have been either undertreated, or they did not respond adequately to the current anti-VEGF treatments and/or could have required a more intensive treatment. In the current study, anti-VEGFs received by patients with nAMD in Korea included ranibizumab, aflibercept, and off-label use of bevacizumab. A treatment that could more effectively provide better disease control without increasing the treatment burden is an unmet need for nAMD management in Korea.

There are several emerging treatments for nAMD that are under investigation, including longer-acting anti-VEGF agents, sustained systems, topical treatments, gene therapy, and molecules targeting novel angiogenesis checkpoints^19,20^. Notably, there are novel agents that can extend treatment intervals up to 12 to 16 weeks^21–23^. The most recent anti-VEGF therapy for nAMD includes brolucizumab, a single-chain antibody fragment inhibitor of all isoforms of VEGF-A^21^. In phase 3 studies, brolucizumab 6 mg demonstrated a better disease control including superior fluid resolution, with > 50% patients treated on a q12w interval after loading up to week 48 versus aflibercept 2 mg dosed q8w^21,22^. However, higher incidence of intraocular inflammation (IOI) was reported in patients treated with brolucizumab (4.7%) compared to aflibercept (< 1%)^21,22^. Following post-marketing reports, a rare safety signal of adverse event of retinal vasculitis (RV) and/ or retinal vascular occlusion (RO) which may result in severe vision loss was identified with brolucizumab.These events typically occured in the presence of IOI^24–26^. Brolucizumab was approved by the Food and Drug Administration in 2019 and European Medicines Agency in 2020 for the treatment of nAMD^27^, and it recently received healthcare reimbursement approval in South Korea.

The VA outcome in this study mirrored the trend observed for retinal fluid status, with the maximum improvement by 3 months after anti-VEGF treatment. In this study, the mean VA gains at month 3 were maintained through 12 months and showed a trend of decline towards 24 months. The VA gain reported in the present study at 12 months (mean + 8.91 letters with 5.54 injections) was relatively higher than in other real-world studies. A systematic review and meta-analysis that assessed the real-world effectiveness of ranibizumab and aflibercept in treatment-naive nAMD patients showed that the VA gain (ETDRS) and injection numbers at 12 months were + 4.24 letters and 5.88 injections with ranibizumab treatment, and + 5.30 letters and 7.10 injections with aflibercept treatment^28^. In the global LUMINOUS study, treatment-naive nAMD patients treated with ranibizumab for 1 year showed a mean VA gain of 3.1 letters with 5.0 injections^29^. A real-world study from US that included 2213 nAMD patients showed that patients treated with anti-VEGFs (ranibizumab, aflibercept, or bevacizumab) for 2 years had a mean change in VA of + 3.1 letters from baseline with 12.1 injections^30^.

This study demonstrates that the retinal fluid status at each time point influenced the VA outcome, whereby patients with absence of retinal fluid had better VA gains than patients with presence of retinal fluid. The same trend was observed for the individual retinal fluid components, including IRF, SRF, and sub-RPE fluid. In some previous studies the presence of SRF was not found to be associated with poor visual outcome^31,32^. Similarly, Core et al., reported that the long-term persisting SRF did not have an impact on VA outcomes through 2 years of follow-up in nAMD patients^33^. However, several recent studies demonstrated contrary results^18,34^. Post hoc analysis of the FLUID study suggested that IRF in the central 1 mm and SRF in the 1 to 6 mm of macular area were negatively associated with best-corrected VA^34^.

Another study with 321 eyes with nAMD showed that absence of IRF or SRF at ≥ 2 clinic visits resulted in a gain of 5 ETDRS letters from baseline, compared with 2 letters gained in eyes with < 2 clinic visits with absence of IRF or SRF^18^. The question of whether SRF can be tolerated is still being debated. In the present study, patients with absence of SRF had overall better VA gain compared with those who had SRF, especially at 12 months after treatment, with 4.52 letters difference in VA gain between the 2 groups. This result may indicate potential negative impact of SRF on visual outcome, implying the need for appropriate SRF control.

We further assessed if the fluid status at month 3 affects the VA outcome at subsequent time points. Compared to patients with presence of retinal fluid, patients with absence of retinal fluid at month 3 achieved slightly better VA gains (~ 1–2 letters) from 3 to 21 months; analyses of SRF and sub-RPE fluid followed a similar pattern. However, in our study, visual outcomes related to the IRF status at month 3 were somewhat different. Closer examination revealed that patients with presence of IRF at month 3 had a relatively lower baseline VA compared with patients with absence of IRF at month 3 (45.32 vs. 55.66 letters) (Fig. S1 and S2). The ceiling effect, whereby patients with higher baseline VA have limited potential to gain more letters, while those with lower baseline VA have little possibility for further loss of vision, may have caused a relatively lower VA gain in the patient subgroup with absence of IRF and vice versa. This finding is in agreement with previous studies, which have reported that the presence of IRF at baseline and during treatment is detrimental to vision^35,36^. It is also in line with the findings from a retrospective 1-year follow-up study of anti-VEGF–treated nAMD eyes showing that presence of IRF was associated with a lower VA at baseline^37^.Patients with presence of IRF at baseline represent a population with more advanced progression of nAMD, highlighting the need to treat IRF aggressively until maximum resolution is achieved^38^. Recently, Core et al. reported the presence of IRF in ≥ 80% of visits through year 2 was closely associated with worse long-term VA and scar development in nAMD patients, confirming the negative impact of persisting IRF on visual prognosis^39^. Our results confirmed that the presence of IRF, SRF, and sub-RPE fluid is associated with a worse VA outcome and suggest that all retinal fluids are pathological. In general, the presence of sub-RPE fluid is not considered to be closely associated with visual prognosis^40^. But few studies have suggested that the sub-RPE fluid can be associated with the prognosis in some circumstances^41,42^. In addition, the incidence of sub-RPE fluid has not been studied in a large Korean cohort. For these reasons, the incidence and clinical significance of sub-RPE fluid were analyzed in this study.

We further assessed the effect of time of absence of retinal fluid on VA outcomes. Patients who were fluid-free for a longer time had relatively better VA gains at both 12 and 24 months, as compared to patients with the shortest time with absence of retinal fluid. This result highlights the importance of long-term fluid control.

In the current study, a similar proportion of patients were treated using the PRN or T&E treatment regimen, with a small proportion of patients adopting an approach of a combination of both regimens. The number of injections and visits for treatment-naive patients with nAMD was 5.54 and 9.51, respectively, in year 1, and 3.48 and 7.10, respectively, in year 2. This was consistent with the real-world ranibizumab treatment pattern for the Korean patient population with nAMD in the LUMINOUS study, in which treatment-naive patients received 5.2 injections and 9.2 monitoring visits by year 1^43^. The number of injections and visits was relatively higher than that in real-world studies conducted in other regions^44–46^ but was relatively lower than that in a study conducted in the US^47^. By 12 months, the patients achieved a VA improvement of + 8.1 letters and CST reduction of 106.90 µm. The results were comparatively better than those from other real-world studies for ranibizumab and aflibercept^28,30,48^.

Overall, the findings of this study are clinically meaningful as they reflect the actual clinical settings in South Korea and provide insights to fluid management strategies. The demographics and clinical characteristics of the study population were consistent with that reported in previous real-world studies from Korea^6,43^. Further, the study included a large sample population of 600 nAMD patients, adding strength to the findings.

Limitations include the retrospective nature of the study. The study cohort was heterogeneous compared to clinical trials. In addition, patients who received ranibizumab, aflibercept, or bevacizumab were pooled in the same cohort, which also included patients who had received two or more different anti-VEGF agents during treatment. Since efficacy might differ amidst the different anti-VEGF agents used, this serves as a major limitation of the study. Decisions regarding treatment regimens and schedule were made based on the physicians’ discretion (PRN or T&E and continuation/discontinuation/switch of treatment). Assessment variables, such as collection and interpretation of retinal images, may also have differed based on the institutions’ practice. Furthermore, the present study evaluated the influence of each fluid compartment on visual outcome separately. For this reason, we could not evaluate the influence of co-existing two or more fluid compartments. In addition, the difference in the impact of different fluid locations (foveal vs extrafoveal) was not evaluated. Lastly, since the current study focused on real-world anti-VEGF treatment patterns, we did not include safety data, which are well-known and reported in the prior literature.

In conclusion, our study found that a high proportion of Korean patients with nAMD on currently available anti-VEGF therapies had reported presence of retinal fluid at year 1 and year 2. Patients who achieved early fluid-free status following anti-VEGF treatment and those who had a longer time of absence of retinal fluid show a trend toward better visual outcomes. New and emerging anti-VEGF therapies that can provide sustained disease control with longer duration of action could lead to improved outcomes and address the high treatment burden for patients requiring more aggressive treatment, thus potentially fulfilling the unmet needs of patients with nAMD in Korea.

## Methods

### Study design and treatment

The PROOF study was a retrospective chart review of patients with nAMD who received their first anti-VEGF treatment between January 2017 and March 2019 in 10 ophthalmology clinics across South Korea. The primary objective of the study was to evaluate the presence of retinal fluid in patients with nAMD after 1 year of anti-VEGF treatment. The study was approved by institutional review board/independent ethics committee for each participating center.

The study period was between July 1, 2016 and March 31, 2020, which allowed for a 6-month pre-index period and an at least 12-month follow-up period. The index/baseline date was defined as the date of the first anti-VEGF injection in patients’ eyes. Patients were administered anti-VEGF treatment, which included ranibizumab, bevacizumab, or aflibercept; the choice of treatment and the decision to discontinue treatment were at the discretion of the prescribing investigator and the patient.

### Eligibility criteria

Treatment-naive patients with nAMD who (1) had received their first anti-VEGF treatment between January 1, 2017 and March 31, 2019; (2) had at least 1 record of nAMD for diagnosis, treatment indication, or clinical findings on or within 6 months prior to the index date; (3) were ≥ 50 years of age at baseline; (4) had retinal fluid status on OCT captured at baseline and at 1 year of follow-up; and (5) had undergone standard examination procedures, including retinal imaging (eg, fundus photography and OCT) at baseline and follow-up, were included. Patients were excluded if they had received any other anti-VEGF and/or photodynamic therapy in the study eye prior to baseline, had concurrent progressive retinal diseases requiring treatment to prevent vision loss (eg, diabetic macular edema, retinal vein occlusion), were receiving any other treatments such as intravitreal steroids or cataract surgery, or participating in any other clinical trial during the study period.

Based on fluorescein angiography (FA) results, the location of choroidal neovascularization (CNV) was categorized into three: subfoveal (involving the foveal center), juxtafoveal (1–199 µm from the foveal center), and extrafoveal (≥ 200 µm from the foveal center). The CNV lesion composition was also classified based on FA results, according to the previously suggested criteria: predominantly classic (the area of classic CNV was at least 50% of the area of the entire lesion), minimally classic (the area of classic CNV was < 50% of the area of the entire lesion, and occult (lesions without classic CNV component)^49^.

### Endpoints

The primary endpoint was the proportion of patients who had retinal fluid (retinal fluid was defined as either presence of intraretinal fluid [IRF], subretinal fluid [SRF], and/or sub-retinal pigment epithelium [RPE] fluid) following 1 year of anti-VEGF treatment. The secondary endpoints included in this report were the proportion of patients with retinal fluid (IRF, SRF, sub-RPE fluid) following anti-VEGF treatment from baseline to month 24, change in VA and CST from baseline to month 24, change in VA stratified by presence/absence of retinal fluid and time with absence of retinal fluid, number of injections and visits, and treatment regimen during the first and second year of treatment.

Secondary data captured in the medical records were extracted retrospectively as the source of the data. An electronic data capture (EDC) method, provided by LSK Global Pharma Services Co. Ltd., Korea, was used in this study; anonymized patient data from the study site were entered directly into the database by the investigator or investigator’s designee via a web browser to establish the patients’ electronic case report form (eCRF). The EDC system verified the integrity of the project progression by conducting system validation.

### Statistical analyses

The primary analysis was performed in patients who met the inclusion criteria of the study and had a minimum follow-up for a period of 12 months (cohort 1, *n* = 600). The longer-term outcomes up to 24 months were additionally evaluated in a subset of patients from cohort 1 who had a minimum follow-up of 24 months (cohort 2, *n* = 453).

For the primary endpoint of the proportion of patients with retinal fluid during the first year of treatment, precision was calculated based on the following assumptions that at least 70% of the patients have retinal fluid and patients would have at least 1 year of follow-up (*N* > 600). With a sample size of 600, the 2-sided 95% confidence interval (CI) for $\pi$ = 0.7 was estimated to range between 66 and 74%. Under the worst case scenario of $\pi$ = 0.5, the 2-sided 95% CI would range between 46 and 54%.

This study was exploratory in nature, and thus, no comparative analysis was planned. All patients who received any anti-VEGFs for nAMD were pooled in the same cohort, and there was no censoring at the time of switching to another anti-VEGF. In patients who received bilateral treatment, only the first treated eye was analyzed. Descriptive statistics (number of observations, proportions, means and standard deviations [SDs], median and minimum and maximum values, and 2-sided 95% CIs of the mean) were tabulated for the demographic and clinical characteristics and all outcome variables unless otherwise stated. Two-sided 95% CIs were derived for all proportions. No data imputation was performed.

For VA analysis, either the naked eye vision or corrected vision was used for each patient. VA was analyzed in subgroups stratified by (1) absence/presence of retinal fluids at each time point: VA at month *N* was stratified by absence/presence of fluids at month *N*; (2) absence/presence of retinal fluids at month 3: VA at all time points was stratified by absence/presence of fluids at month 3; (3) time with absence of fluid over 1 to 12 months (cohort 1) and 1 to 24 months (cohort 2): time was calculated as the sum of fluid-absence duration (time from the absence of fluid to a day before subsequent presence from day 1 to 360 and day 1 to 720 in days). Patients were divided into quartiles groups based on their time with absence of fluid. Quartile values for cohort 1: lower quartile = 0.0 day, median = 1.0 day, upper quartile = 139.5 days; quartile values for cohort 2: lower quartile = 1.0 day, median = 86.0 days, upper quartile = 288.0 days.

The Wilcoxon signed-rank test was used to assess the difference in VA change between patients with presence and absence of retinal fluid, and in patients with different quartiles of time with absence of retinal fluid. Two-sided *P values* were reported. All analyses were performed by LSK Global Pharma Services Co. Ltd., Korea, using Statistical Analysis System (SAS) version 9.4 software.

### Ethical approval

This study was conducted in accordance with the Guidelines for Good Pharmacoepidemiology Practices issued by the International Society for Pharmacoepidemiology and the Strengthening the Reporting of Observational Studies in Epidemiology (STROBE) guidelines, and adhered to the ethical principles derived from the Declaration of Helsinki. Each center’s institutional review board/independent ethics committee (IRB/IEC) reviewed and approved the study protocol.

### Informed consent

Patients from Samsung Medical Center provided written informed consent, and the IRB/IEC of the other study sites approved the waiver of patient consent. All research adhered to the tenets of the Declaration of Helsinki. No animal subjects were included in this study.

## Supplementary Information

Supplementary Information 1.

Supplementary Information 2.

## Data Availability

The datasets used and/or analyzed during the current study are available from the corresponding author on reasonable request.
