# Supplementary figures and images for: A real-world study assessing the impact of retinal fluid on visual acuity outcomes in patients with neovascular age-related macular degeneration in Korea
Source: Sci Rep. 2022 Aug 19;12:14166. doi: 10.1038/s41598-022-18158-z (PMC9391430; doi:10.1038/s41598-022-18158-z)

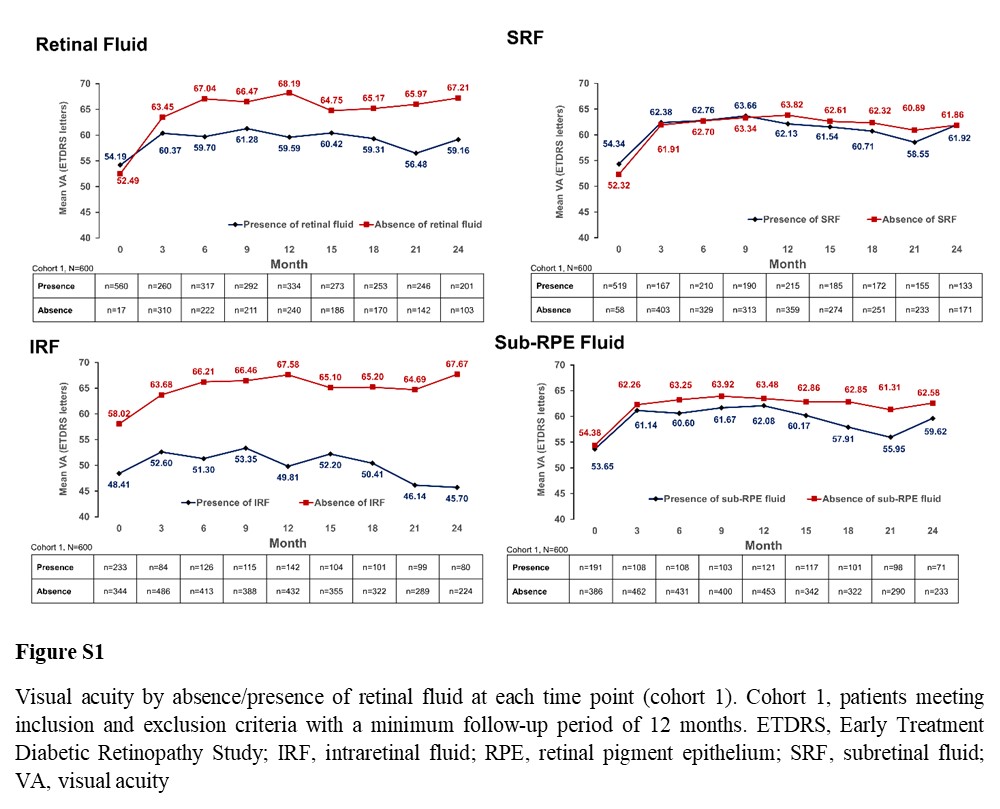

Supplement: Supplementary file 1 — Supplementary Information 1. [file 41598_2022_18158_MOESM1_ESM.tiff]

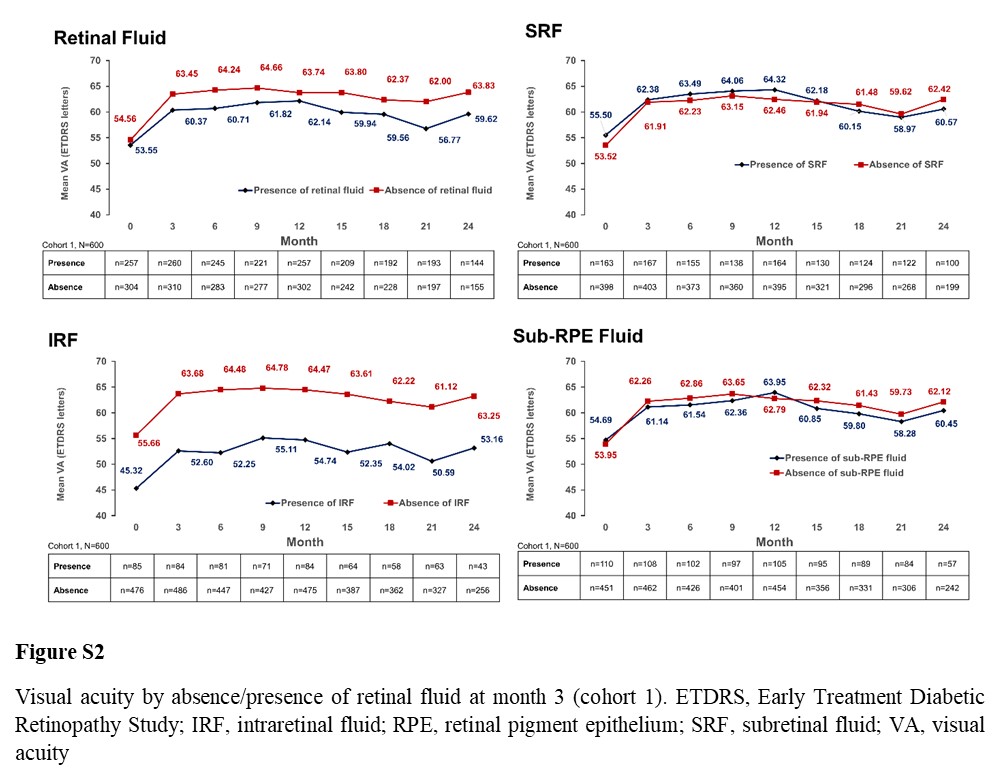

Supplement: Supplementary file 2 — Supplementary Information 2. [file 41598_2022_18158_MOESM2_ESM.tiff]
